# Preliminary Identification of Coping Profiles Relevant to Surrogate Decision Making in the ICU

**DOI:** 10.1371/journal.pone.0166542

**Published:** 2016-11-11

**Authors:** Jorie M. Butler, Eliotte L. Hirshberg, Ramona O. Hopkins, Emily L. Wilson, James F. Orme, Sarah J. Beesley, Kathryn Kuttler, Samuel M. Brown

**Affiliations:** 1 Geriatric Research Education and Clinical Center (GRECC), George E. Wahlen Department of Veterans Affairs Medical Center, Salt Lake City, Utah, United States of America; 2 Department of Internal Medicine, Division of Geriatrics, University of Utah, Salt Lake City, Utah, United States of America; 3 Center for Humanizing Critical Care, Intermountain Healthcare, Murray, Utah, United States of America; 4 Department of Medicine, Pulmonary and Critical Care Division, Intermountain Medical Center, Murray, Utah, United States of America; 5 Department of Internal Medicine, Division of Pulmonary Medicine, University of Utah, Salt Lake City, Utah, United States of America; 6 Department of Pediatrics, Division of Critical Care, University of Utah, Salt Lake City, Utah, United States of America; 7 Psychology Department and Neuroscience Center, Brigham Young University, Provo, Utah, United States of America; 8 Homer Warner Center for Informatics Research, Murray, Utah, United States of America; University of Akron, UNITED STATES

## Abstract

**Objective:**

The Intensive Care Unit (ICU) is a stressful environment for families of critically ill patients and these individuals are at risk to develop persistent psychological morbidity. Our study objective was to identify individual differences in coping with stress and information presentation preferences of respondents exposed to a simulated ICU experience.

**Methods:**

Participants were recruited from a university and two community populations. Participants completed questionnaires that measured demographic information and characteristics that may be relevant to an individual’s ICU experience. Quality of life was measured by the EQ-5D, personality dimensions were examined with the abbreviated Big Five inventory, coping with stress was assessed with Brief COPE. Shared decision making preferences were assessed by the Degner Control Preferences Scale (CPS) and information seeking style was assessed with the Miller Behavioral Style Scale (MBSS). Social support was examined using an abbreviated version of the Social Relationship Index. Participants also completed a vignette-based simulated ICU experience, in which they made a surrogate decision on behalf of a loved one in the ICU.

**Results:**

Three hundred forty-three participants completed the study. Three distinct coping profiles were identified: adaptive copers, maladaptive copers, and disengaged copers. Profiles differed primarily on coping styles, personality, quality of their closest social relationship, and history of anxiety and depression. Responses to the simulated ICU decision making experience differed across profiles. Disengaged copers (15%) were more likely to elect to refuse dialysis on behalf of an adult sibling compared to adaptive copers (7%) or maladaptive copers (5%) (p = 0.03). Notably, the MBSS and the CPS did not differ by coping profile.

**Conclusion:**

Distinct coping profiles are associated with differences in responses to a simulated ICU experience. Tailoring communication and support to specific coping profiles may represent an important pathway to improving ICU experience for patients and families.

## Introduction

The intensive care unit (ICU) can be a difficult and stressful environment for families of patients to navigate. During an ICU stay, families confront and process potentially life-changing events and emotionally charged information in a setting of deep uncertainty and substantial risk of death for their loved one [1–3]. The ICU experience is multi-faceted, including worry over possible mortality in the context of sleep deprivation, emotional exhaustion, and circumstances that may change rapidly. This high-stakes, high-stress setting can trigger negative emotions including fear, anger, and fatigue [4, 5]. Family members can experience psychological morbidity including acute stress, anxiety, depression, and posttraumatic stress disorder (PTSD) during and after the ICU experience [3, 6–8]. Persistent psychological morbidity after an ICU hospitalization is part of a syndrome called post-intensive care syndrome-Family (PICS-F) [9]. The causes of persistent psychological morbidity are likely multifactorial and include pre-existing risk factors, information needs of families, and medical decision-related stress [1, 3].

Key components of the ICU experience for families include the medical decisions required during an ICU stay. Decision related stress may be a modifiable risk factor for persistent psychological morbidity. Many ICU patients are too ill to participate in decision making [10], therefore the burden of medical decision making commonly falls on family members, termed “surrogate decision makers” [7]. Ideally, information should be reciprocally communicated as part of shared decision making between surrogate decision makers and ICU providers. The ICU is a challenging place for shared decision making because the wishes of the patient may be difficult to discern, many of the decisions are made under time pressures, and the outcomes (e.g. mortality relating to stopping treatment) can have intense practical, physical and emotional significance [11, 12]. Surrogate decision making can be stressful and distressed surrogates may find information difficult to process [13]. Information-related concerns reported by surrogate decision makers include unwanted information, lack of clear information, and not enough time allowed for information to be received [3, 14]. Perceived inadequacy of information sharing in the ICU is associated with adverse post-ICU stress reactions [3]. In contrast, satisfaction with communication in the ICU is associated with less psychological morbidity [15, 16]. Aligning communication and shared decision making in the ICU to surrogate preferences and vulnerabilities may ameliorate psychological morbidity for family members during an ICU stay and improve satisfaction with the ICU experience [14].

The scope of medicine has expanded to include family experience [17, 18], and consequently personalized medicine must respect and adapt to individual psychological attributes of family members. In addition to decisional preferences, surrogates enter the ICU with unique characteristics that may affect their risk for psychological morbidity both during and after the ICU [17, 19, 20]. In trauma-exposed populations, individual attributes, such as neuroticism and history of anxiety have been associated with the development of PTSD [19, 21, 22], suggesting that prospective identification of attributes associated with negative stress reactions is possible. Identifying such attributes at the outset of an ICU experience is an important step in advancing patient- and family- centered care in acute care environments [23, 24]. Personalized support strategies in the ICU may help to ameliorate the morbidities of PICS-F [20].

Our study objective was to identify individual differences in coping with stress, information presentation preferences, and social relationships in association with a simulated ICU experience. We hypothesized that individual attributes and decision making preferences relevant to the ICU would predict both decisions made during a simulated ICU experience and the overall rating of the quality of shared decision making. This study was intended to serve as the initial step in establishing coping profiles among surrogate decision makers in the ICU. The ultimate goal is to inform the design and testing of interventions that tailor support and communication to family members in the ICU

## Materials and Methods

### Study Population

Participants were recruited through the Department of Psychology subject pool at the University of Utah, the Osher Institute for Lifelong Learning at the University of Utah (a community organization for members 50 years of age and older), and Community Faces of Utah (a Utah-based, community-university-health department collaborative that includes leaders of five ethnically diverse community organizations). Inclusion criteria included participants 18 years of age or older, able to read and write in English, and an active member of the organizations from which the sample was recruited–University of Utah, Osher Lifelong Learning Institute, or Community Faces of Utah. There were no specific exclusion criteria.

### Ethical Considerations

Institutional Review Boards from University of Utah and Intermountain Medical Center approved the study and all procedures. An informed consent cover letter was included with all questionnaires, and survey completion implied consent. As compensation, undergraduates received course extra credit. Osher Institute and Community Faces of Utah participants received a $25 gift card.

### Questionnaires

We administered questionnaires to assess demographics, personality characteristics, shared decision making preferences, information seeking style, coping styles, and social support. We chose these domains for investigation because they had well-validated measures, had been associated with adverse stress responses to ICU hospitalization, or had good face validity for their potential relevance to the development of communication and support strategies for families of ICU patients.

#### Demographic characteristics

Demographic characteristics included participant age, sex, ethnicity, years of educational attainment, experience as a patient or loved one in the hospital and/or ICU, and identification with religion (e.g., *Buddhism*, *Christianity*, *Islam* as well as *spiritual but not religious* or *other*) were collected. The importance of religion was assessed using the following questions: *How important is religion in your daily life*? with answers ranging from (1) *not at all* to (4) *extremely*: and the frequency of religious service attendance *How frequently do you attend religious services*?. History of anxiety and depression was assessed by asking *Have you ever been treated for anxiety by a physician or mental health provider*?. A parallel question was asked about past treatment for depression.

#### Quality of life

The EQ-5D-3L[25] is a widely used, reliable, and validated instrument [26] that evaluates quality of life across five dimensions including mobility, self-care, usual activities, pain/discomfort and anxiety/depression. Responses are measured on a 3-point scale from (1) *no problems* to (3) *a lot of problems*. These responses are mapped onto US health utilities. Lower utilities indicate lower quality of life [27].

#### Personality dimensions

The abbreviated 10-item Big Five Inventory (BFI-10) [28] was used to assess personality dimensions including openness to new experiences, conscientiousness, extraversion, agreeableness, and neuroticism. Higher scores indicate a greater degree of the specific dimension This measure has been shown to be reliable and valid in other populations [28].

#### Coping with stress

Participants were asked to think of the most stressful situation they had experienced during the past five years, describe it, and indicate how they coped with it. The valid and reliable Brief COPE [29] includes two questions per dimension across 14 dimensions, including active coping (*I’ve been concentrating my efforts on doing something about the situation I’m in)* using emotional support (*I’ve been getting comfort and understanding from someone)* and venting (*I’ve been expressing my negative feelings*) and has been used in many health-related settings. Higher scores indicated greater use of a given coping strategy.

#### Medical decision control preferences

The preferred level of engagement in shared decision making was assessed by the Degner Control Preferences Scale [30] with the question. *How would you prefer to make decisions with your doctor*?. Response choices ranged from highly autonomous decision making (*I prefer to make the final selection about which treatment I will receive* (1)) to highly deferential (*I prefer to leave all decisions regarding my treatment to my doctor* (5)), with equally shared decision making in the middle (3).

#### Information seeking styles under stress

The Miller Behavioral Style Scale (MBSS) [31] determines whether people are primarily “monitors” (avid information seekers) or “blunters” (avoiding information through strategies such as self-distraction) based on their responses to hypothetical stressful situations. The number of scenarios presented was reduced from four to three to decrease respondent burden [32, 33]. Responses associated with monitoring and blunting are summed across the three situations. Higher scores indicate a preference for monitoring over blunting.

#### Social support

The Social Relationship Index (SRI) [34] measures positivity and negativity in social relationships. The SRI was modified to a self-administered version and only information regarding the relationship rated most important to the respondent was used for analysis. Given the reliance on a single patient-designated surrogate in many clinical situations [35] and the role for emotional closeness between surrogate and patient in determining the accuracy of a surrogate decision [36] we chose the most important relationship as the focus for this study. Participants rated support from their most important relationship on dimensions of importance, helpfulness, and negativity on a 1–6 point interval scale with 1 indicating low levels and 6 indicating high levels.

### Outcome Measures

We developed a vignette-based simulated ICU experience (see Appendix A, in S1 File). In the vignette, respondents were asked to make decisions on behalf of an adult sibling with whom they have a close relationship. Two adult intensivists, a pediatric intensivist, a health psychologist and a cognitive psychologist who specializes in ICU outcomes generated the multi-part simulated ICU decision making experience, which required surrogate decision making. Multiple members of the research team have conducted vignette-based research studies. The simulated ICU experience was developed iteratively by the team and pilot tested for clarity and time of administration. In the simulated ICU experience, respondents were asked to imagine being the close sibling of a 68-year old female patient with obesity and diabetes mellitus. The patient had expressed to the respondent that she did not wish to be “kept alive as a vegetable.” The patient was admitted to the ICU with severe pneumonia that required mechanical ventilation. The participants were informed of a decline in the patient’s clinical condition and asked to make a critical decision about the patient’s care, whether to accept or reject kidney dialysis (details available in Appendix A in S1 File). This decision was chosen as the focus because dialysis is a common end of life choice and often requires different shared decision making approaches [37]. After completing the simulated ICU decision making task, the participants rated the adequacy of shared decision making using the CollaboRATE instrument [38, 39], which measures the perception of efforts made to assure shared decision making on a 10-point interval scale. The CollaboRATE has been validated in previous simulated doctor-patient encounters [39].

### Statistical Methods

Descriptive statistics were calculated (by recruitment cohort and by coping profile) for variables as mean ± standard deviation (SD), median (interquartile range; IQR), or proportion (%) as appropriate. P-values were estimated by Kruskal Wallis tests for continuous variables and Chi-square tests (or Fisher’s exact tests when cell counts were low) for proportions.

Cluster analysis, employing the well-established *k*-means technique [40, 41], was used to identify groupings of participants based on their responses to survey instruments. Cluster analysis groups observations across variables based on similarity, as defined by a measure of distance (in this case Euclidian distance). We determined the optimal value of *k* (the number of clusters) to be the value associated with the lowest misclassification rate. To assist visualization of cluster separation, we displayed clusters on a biplot of the first two principal components from a principal components analysis of the underlying instruments. We also explored differences in constituent instruments by cluster, using Chi-square tests for proportions or Kruskal-Wallis tests, where appropriate. For our outcome analysis we compared clusters on whether respondents chose kidney dialysis and how the respondent scored the adequacy of shared decision making, using the standard “top score” method for scoring CollaboRATE [38, 39].

Our primary analysis was of the determination of different coping profiles, which were identified as clusters based on responses to the instrument battery. Secondarily, we explored differences in decision making (i.e., the decision to proceed with dialysis) and perceived adequacy of shared decision making (CollaboRATE score) by coping profile.

In a further exploratory analysis, we investigated the relevance of prior ICU experience (either personal or family) to the outcome analysis. We evaluated whether ICU experience was associated with outcomes and whether the associations by coping profile differed by ICU experience. All analyses were performed within the R statistical computing environment [42].

## Results

### Demographics

A total of 414 respondents (296 University of Utah undergraduates, 69 participants from the Osher Institute, and 49 participants from Community Faces of Utah) participated in the study. Of these, 343 (83%) participants (259 from University of Utah undergraduates, 48 from Osher, and 36 from CFU) had complete data for both the instrument battery and the simulated decision making and were included in the final sample. Demographic characteristics of respondents are summarized in Table 1.

**Table 1:** Demographic Characteristics of Respondents by Recruitment Cohort.

	Combined Group (n = 343)	Undergraduates (n = 259)	Osher (n = 48)	CFU (n = 36)	p value
**Age; median (IQR)**	22 (20–37)	21 (19–24)	68 (64–73)	41 (34–62)	**<0.001**
**Female; n (%)**	233 (68%)	181 (70%)	34 (71%)	18 (50%)	0.081
**Ethnicity; n (%)**					**<0.001**
African-American	17 (5%)	3 (1%)	1 (2%)	13 (36%)	
Asian	22 (6%)	20 (8%)	2 (4%)	0 (0%)	
Latino/Hispanic	33 (10%)	24 (9%)	1 (2%)	8 (22%)	
Native American	8 (2%)	5 (2%)	0 (0%)	3 (8%)	
Pacific Islander	9 (3%)	1 (< 1%)	0 (0%)	8 (22%)	
White/Caucasian	245 (71%)	200 (77%)	44 (92%)	1 (3%)	
Other	8 (2%)	5 (2%)	0 (0%)	3 (8%)	
**Education; n (%)**					**<0.001**
Some schooling	4 (1%)	0 (0%)	0 (0%)	4 (11%)	
High school graduate (or equivalent)	16 (5%)	12 (5%)	0 (0%)	4 (11%)	
Some college, less than one year	44 (13%)	42 (16%)	1 (2%)	1 (3%)	
One or more years of college, no degree	133 (39%)	117 (45%)	4 (8%)	12 (33%)	
Associate Degree	75 (22%)	70 (27%)	2 (4%)	3 (8%)	
Bachelor’s Degree	39 (11%)	14 (5%)	17 (35%)	8 (22%)	
Master’s Degree	15 (4%)	1 (< 1%)	13 (27%)	1 (3%)	
Professional Degree	6 (2%)	0 (0%)	6 (13%)	0 (0%)	
Doctorate Degree	5 (2%)	1 (< 1%)	4 (8%)	0 (0%)	
**Religious Affiliation; n (%)**					**<0.001**
Buddhist	8 (2%)	5 (2%)	3 (6%)	0 (0%)	
Catholic	36 (11%)	28 (11%)	5 (10%)	3 (8%)	
Christian (not Catholic, Protestant, or LDS)	36 (11%)	29 (11%)	2 (4%)	5 (14%)	
Hindu	0 (0%)	0 (0%)	0 (0%)	0 (0%)	
Jewish	1 (< 1%)	1 (< 1%)	0 (0%)	0 (0%)	
LDS/Mormon	103 (30%)	82 (32%)	10 (21%)	11 (31%)	
Muslim	4 (1%)	4 (2%)	0 (0%)	0 (0%)	
Native American	5 (1%)	2 (1%)	0 (0%)	3 (8%)	
Protestant (such as Baptist, Methodist, or Presbyterian)	26 (8%)	6 (2%)	9 (19%)	11 (31%)	
Spiritual but not religious	50 (15%)	43 (17%)	6 (13%)	1 (3%)	
Nothing in particular	66 (19%)	55 (21%)	10 (21%)	1 (3%)	
Other	7 (2%)	4 (2%)	2 (4%)	1(3%)	

Totals may not equal 100% given missing data for some demographic variables. A Bonferroni adjusted significance level of 0.01 was used for all comparisons. CFU, Community Faces of Utah; IQR, Interquartile range; LDS, Church of Jesus Christ of Latter-day Saints.

### Missing Data

There were 28 cases (7%) with missing data on one or more predictor variables. Respondents with complete data versus incomplete data did not differ by sex, educational attainment, or history of anxiety or depression. Participants with incomplete data were somewhat older (median age 27 vs. 22 years, p = 0.01).

To determine whether patterns of missingness affected our analyses, we compared the cluster analysis that identified coping profiles using complete cases with the cluster analysis using imputed data (values were imputed using a simple, multivariable imputation technique).Results of the sensitivity analysis demonstrated no difference in the pattern of findings so only complete cases were used in our primary analysis (details are provided in S2 File).

### Descriptive Results

Two-thirds of participants (69%) had a relative who had been hospitalized in the ICU. More than half (54%) had experience with hospitalization as a patient and, of these, 24% (n = 44) reported having been hospitalized in an ICU (median length of time since admission, 5 years). Personal recollection of the ICU experience was relatively positive, with an overall mean of 3.8 on a 5-point Likert-style item (5 representing “quite positive”) and recollections of a relative’s ICU admission was similarly positive (mean 4.0 on the same Likert-style item). A substantial minority (26%) of participants reported a history of anxiety and 30% had been treated for depression.

The content of the participants’ most stressful event in the past 5 years was heterogeneous, with 25% of individuals reporting coping with medical problems or death of a loved one. Other stressful events included problems with romantic relationships (12%), school-related problems (14%), and major life changes (11%). The mean rating of the reported stressful event was 3.9 across the sample (SD = 0.7) on an ordinal scale from 1 (*not stressful at all*) to 5 (*most stressful experience imaginable*).

### Cluster Analysis

**Fig 1:**
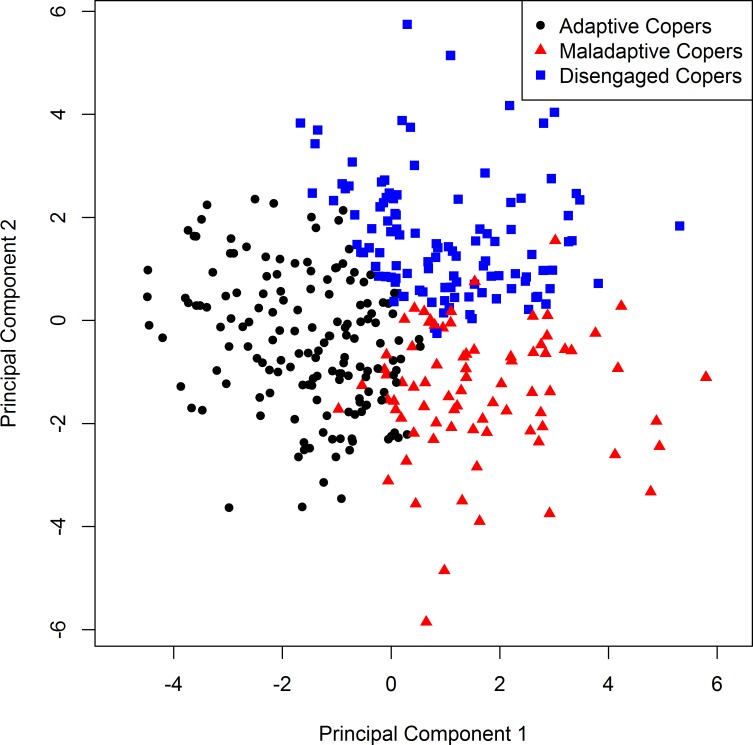
Coping ProfileSeparation. Participants are represented by a different colored shape for each coping profile. Black circles represent the Adaptive Copers’, red triangles represent the Maladaptive Copers’, and blue squares represent the Disengaged Copers’.

Cluster analysis identified a 3-cluster solution, based on misclassification rates from discriminant analysis (lowest for the 3-cluster solution: 8%). Fig 1 displays the three clusters on a biplot of the first two principal components; this biplot demonstrates good separation of the clusters. After discussion and consensus among the authors with the goal of selecting parsimonious terms on the basis of individual differences in attributes we chose to describe the three clusters as coping profiles, specifically “adaptive copers,” “maladaptive copers,” and “disengaged copers.” Table 2 displays demographic information by coping profile.

**Table 2:** Demographics by Coping Profile.

	Adaptive Copers (n = 155)	Maladaptive Copers (n = 78)	Disengaged Copers (n = 110)	p value
**Age; median (IQR)**	24 (20–42)	21 (19–25)	23 (20–40)	**0.002**
**Female; n (%)**	115 (74%)	57 (73%)	61 (55%)	**0.003**
**Ethnicity; n (%)**				0.688
African-American	7 (5%)	4 (5%)	6 (5%)	
Asian	8 (5%)	3 (4%)	11 (10%)	
Latino/Hispanic	13 (8%)	10 (13%)	10 (9%)	
Native American	4 (3%)	1 (1%)	3 (3%)	
Pacific Islander	7 (5%)	0 (0%)	2 (2%)	
White/Caucasian	111 (72%)	58 (74%)	76 (69%)	
Other	4 (3%)	2 (3%)	2 (2%)	
**Education; n (%)**				0.212
Some schooling	1 (1%)	0 (0%)	3 (3%)	
High school graduate (or equivalent)	8 (5%)	2 (3%)	6 (5%)	
Some college, less than one year	14 (9%)	17 (22%)	13 (12%)	
One or more years of college, no degree	60 (39%)	32 (41%)	41 (37%)	
Associate Degree	38 (25%)	17 (22%)	20 (18%)	
Bachelor’s Degree	20 (13%)	5 (6%)	14 (13%)	
Master’s Degree	8 (5%)	1 (1%)	6 (5%)	
Professional Degree	3 (2%)	0 (0%)	3 (3%)	
Doctorate Degree	1 (1%)	2 (3%)	2 (2%)	
**Religious Affiliation; n (%)**				0.006
Buddhist	5 (3%)	1 (1%)	2 (2%)	
Catholic	17 (11%)	10 (13%)	9 (8%)	
Christian (not Catholic, Protestant, or LDS)	13 (8%)	10 (13%)	13 (12%)	
Hindu	0 (0%)	0 (0%)	0 (0%)	
Jewish	1 (1%)	0 (0%)	0 (0%)	
LDS	55 (35%)	24 (31%)	24 (22%)	
Muslim	3 (2%)	0 (0%)	1 (1%)	
Native American	3 (2%)	1 (1%)	1 (1%)	
Protestant (such as Baptist, Methodist, or Presbyterian)	14 (9%)	2 (3%)	10 (9%)	
Spiritual but not religious	25 (16%)	13 (17%)	12 (11%)	
Nothing in particular	14 (9%)	17 (22%)	35 (32%)	
Other	4 (3%)	0 (0%)	3 (3%)	
**ICU Experience**				
ICU admission (personal); n (%)	24 (15%)	11 (14%)	9 (8%)	0.200
Years since ICU admission; median (IQR)	6 (3–14)	3 (2–7)	6 (2–10)	0.471
Experience with ICU**; mean ± SD	4.1 ± 0.9	3.8 ± 1.2	3.0 ± 1.1	0.056
Relative ICU admission; n (%)	117 (75%)	53 (68%)	67 (61%)	0.039
Relative experience with ICU**; mean ± SD	4.1 ± 1.0	4.1 ± 0.8	3.7 ± 1.0	0.057

A Bonferroni adjusted significance level of 0.005 was used for all comparisons. IQR, Interquartile range; LDS, Church of Jesus Christ of Latter-day Saints; ICU, Intensive Care Unit; SD, Standard Deviation.

** Rated on a scale from 1 = Terrible to 5 = Quite Positive

**Fig 2:**
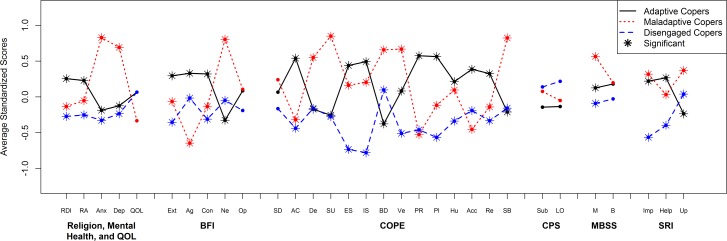
Average Standardized Coping Profile Component Scores. The dots represent the average standardized scores for each instrument subscale. Black solid lines represent Adaptive Copers, red dashed lines represent Maladaptive Copers, and blue long dashed lines represent Disengaged Copers. Abbreviations: Religion, Mental Health, and QOL, Religion, Mental Health, and Quality of Life; RDI, Religion Daily Importance; RA, Religious Attendance; Anx, Anxiety; Dep, Depression; QOL, Quality of Life; BFI, Big Five Inventory; Ext, Extraversion; Ag, Agreeableness; Con, Conscientiousness; Ne, Neuroticism; Op, Openness; COPE, Brief COPE; SD, Self-distraction; AC, Active Coping; De, Denial; SU, Substance Use; ES, Emotional Support; IS, Instrumental Support; BD, Behavioral disengagement; Ve, Venting; PR, Positive Reframing; Pl, Planning; Hu, Humor; Acc, Acceptance; Re, Religion; SB, Self-blame; CPS, Degner Control Preference Scale; Sub, Subject; LO, Loved One; MBSS, Miller Behavioral Style Scale; M, Monitoring; B, Blunting; SRI, Social Relationship Index; Imp, Importance; Help, Helpfulness; Up, Upsetting.

Fig 2 represents the average standardized scores for each instrument and its subscales. Means and standard deviations for scales and subscales for each instrument are reported in Table 3. Notably, coping profile membership did not differ by the respondents’ prior ICU experience. Nor did the coping profile members differ on the Control Preferences Scale. As assessed by visual inspection and misclassification rates, coping profile structure did not vary by recruitment cohort (data not shown).

**Table 3:** Descriptive Characteristics by Coping Profile.

	Overall (n = 343)	Adaptive Copers (n = 155)	Maladaptive Copers (n = 78)	Disengaged Copers (n = 110)	p value
	**Mean ± SD**				
**Religion**					
Daily Importance	2.2 ± 1.1	2.5 ± 1.2	2.1 ± 1.1	1.9 ± 1.0	**<0.001**
Attendance	4.4 ± 2.8	5.0 ± 2.9	4.2 ± 2.6	3.6 ± 2.7	**<0.001**
**Quality of Life (EQ-5D)**	0.89 ± 0.08	0.90 ± 0.08	0.86 ± 0.07	0.90 ± 0.08	0.062
**Big Five Inventory-10**					
Extroversion	3.2 ± 0.9	3.5 ± 0.9	3.2 ± 0.9	2.9 ± 0.9	**<0.001**
Agreeableness	3.6 ± 0.8	3.9 ± 0.7	3.1 ± 0.8	3.6 ± 0.8	**<0.001**
Conscientiousness	3.9 ± 0.8	4.2 ± 0.7	3.8 ± 0.7	3.7 ± 0.8	**<0.001**
Neuroticism	2.8 ± 0.9	2.5 ± 0.8	3.6 ± 0.9	2.8 ± 0.8	**<0.001**
Openness	3.7 ± 0.9	3.7 ± 0.8	3.8 ± 0.8	3.5 ± 0.9	0.046
**Degner Control Preferences Scale**					
Subject	2.7 ± 1.1	2.5 ± 0.9	2.8 ± 1.1	2.8 ± 1.2	0.220
Loved One	2.6 ± 1.2	2.4 ± 1.0	2.5 ± 1.3	2.8 ± 1.3	0.034
**Brief COPE**					
Self Distraction	3.0 ± 0.8	3.0 ± 0.8	3.2 ± 0.7	2.8 ± 0.8	0.017
Active Coping	3.2 ± 0.7	3.6 ± 0.5	3.0 ± 0.6	2.9 ± 0.7	**<0.001**
Denial	1.6 ± 0.8	1.5 ± 0.7	2.1 ± 1.1	1.5 ± 0.7	**<0.001**
Substance Use	1.5 ± 0.8	1.3 ± 0.5	2.2 ± 1.2	1.2 ± 0.5	**<0.001**
Emotional Support	3.0 ± 0.8	3.3 ± 0.7	3.1 ± 0.7	2.4 ± 0.8	**<0.001**
Instrumental Support	2.9 ± 0.9	3.3 ± 0.7	3.1 ± 0.7	2.2 ± 0.8	**<0.001**
Behavioral Disengagement	1.6 ± 0.7	1.3 ± 0.5	2.0 ± 0.8	1.6 ± 0.7	**<0.001**
Venting	2.5 ± 0.8	2.6 ± 0.8	3.1 ± 0.6	2.1 ± 0.7	**<0.001**
Positive Reframing	3.0 ± 0.9	3.5 ± 0.6	2.5 ± 0.9	2.6 ± 0.8	**<0.001**
Planning	3.3 ± 0.7	3.7 ± 0.4	3.2 ± 0.7	2.9 ± 0.7	**<0.001**
Humor	2.1 ± 1.0	2.3 ± 1.0	2.2 ± 1.0	1.8 ± 0.8	**<0.001**
Acceptance	3.2 ± 0.7	3.5 ± 0.5	2.9 ± 0.7	3.1 ± 0.7	**<0.001**
Religion	2.4 ± 1.2	2.8 ± 1.1	2.2 ± 1.2	2.0 ± 1.1	**<0.001**
Self Blame	2.4 ± 1.0	2.2 ± 0.9	3.2 ± 0.8	2.2 ± 1.0	**<0.001**
**Miller Behavioral Style Scale**					
Monitoring	5.2 ± 2.7	5.1 ± 2.5	6.3 ± 2.7	4.7 ± 2.7	**<0.001**
Blunting	3.1 ± 2.1	3.2 ± 2.1	3.2 ± 2.2	2.8 ± 1.9	0.233
**Social Relationship Index**					
Important	5.9 ± 0.4	6.0 ± 0.2	6.0 ± 0.1	5.6 ± 0.6	**<0.001**
Helpful	5.1 ± 1.0	5.4 ± 0.8	5.2 ± 0.9	4.8 ± 1.2	**<0.001**
Upsetting/Negativity	2.0 ± 1.0	1.8 ± 0.9	2.4 ± 1.1	2.1 ± 1.1	**<0.001**
	**N (%)**				
**History of Anxiety or Depression**					
Anxiety Treatment	90 (26%)	28 (18%)	49 (63%)	13 (12%)	**<0.001**
Depression Treatment	103 (30%)	36 (23%)	47 (60%)	20 (18%)	**<0.001**

A Bonferroni adjusted significance level of 0.0016 was used for all comparisons. SD, Standard Deviation.

#### Adaptive copers

Of the adaptive copers (n = 155), 74% were female with age ranging from 18 to 83 years with a median (IQR) of 24 (20–42) years. Adaptive copers were relatively high in extraversion, agreeableness, openness, and conscientiousness, and low on neuroticism. Participants in this coping profile considered religion important and attended religious services regularly (about once per month), and 18% had a history of anxiety treatment whereas 23% had a history of depression treatment. Adaptive copers also reported higher use of active coping strategies as well as receiving emotional and instrumental support from others. They used positive reframing, planning, humor, acceptance, and religion when coping. By contrast, these adaptive copers reported low levels of behavioral disengagement and self-blame in coping. Adaptive copers reported a mid-level of “monitoring” behaviors in response to stress, on the MBSS. The most important personal relationship for adaptive copers was relatively low in negativity and was high in importance and helpfulness relative to other coping profiles.

#### Maladaptive copers

Of maladaptive copers (n = 78) 73% were female with age ranging from 18 to 69 and median (IQR) of 21 (19–25) years. In terms of personality traits, they demonstrated high neuroticism and low agreeableness. A majority (63%) reported a history of anxiety treatment and 60% reported a history of depression treatment. Participants in this coping profile reported coping with a stressful event by using substances, denial, behavioral disengagement, and venting. Maladaptive copers were less likely to use acceptance for coping and reported higher levels of self-blame. They reported relatively high levels of “monitoring” on the MBSS. The most important relationship among maladaptive copers was high in importance and high in negativity relative to respondents in other coping profiles.

#### Disengaged copers

Disengaged copers were 55% female with an age range of 18 to 84 years and median (IQR) age of 23 (20–40) years. The disengaged copers were relatively low in extraversion and mid-range in neuroticism. The importance of religion and religious attendance were low for this group, and they were less likely than those in other coping profiles to report a history of anxiety (12%) or depression (18%) treatment. When coping with stressful events, disengaged copers were less likely to receive emotional or instrumental support; they were also less likely to use planning, humor, or religion as a means of coping with stress. Disengaged copers were relatively low “monitors” for information on the MBSS. The most important relationship of disengaged copers was rated fairly low in importance and helpfulness and mid-range in negativity relative to other coping profiles. Descriptive results by coping profile are depicted in Tables 2 and 3.

*Differences among coping profiles in response to a simulated ICU decision making experience*. Responses to the simulated ICU decision making experience differed across coping profiles. Disengaged copers (15%) were more than twice as likely to refuse dialysis on behalf of an adult sibling compared to adaptive copers (7%) or maladaptive copers (5%) (p = 0.03).

Respondents also differed in their ratings of the quality of shared decision making within the simulated experience, as measured by CollaboRATE. Among adaptive copers, 15% of the respondents selected the highest score for shared decision making (suggesting high satisfaction with shared decision making) as compared to only 5% of maladaptive copers, and 4% of disengaged copers (p = 0.003).

*Analysis exploring the relevance of prior ICU experience*. To explore implications of the prior ICU experiences of participants in our sample, we examined responses to the simulated experience by coping profile and ICU experience. Participants who had a prior ICU experience differed in likelihood of reporting the highest score (score of 10) on CollaboRATE: among respondents who had a prior ICU experience, 18% of adaptive copers, 6% of maladaptive copers, and 2% of disengaged copers reported highest score (p = 0.002). Among respondents without prior ICU experience, there were no differences among coping profiles in rates of highest score on the CollaboRATE. The differences by coping profile observed in terms of decision making (i.e., the decision to refuse dialysis) did not change when comparing individuals with and without prior ICU experience.

## Discussion

We found that distinct coping profiles were associated with different responses to a simulated ICU experience in a community sample. There were differences by coping profile in surrogate decision making (disengaged copers were more likely to refuse treatment on behalf of an adult sibling) and ratings of the ICU experience (adaptive copers rated shared decision making experience more positively). These findings suggest that personal attributes as well as explicit beliefs may affect surrogate decision making. This is consistent with prior observations that surrogate decision makers tend to make decisions more compatible with their own views on life rather than the patients’ [35]. Our findings support ongoing concerns regarding surrogate decision making, providing greater insight into the established limitations of this practice [35, 43].

An important contribution of this study is that we used a simulated experience and were therefore able to offer an identical simulated ICU experience to all participants. Differences in ratings of shared decision making and/or experience were thought to reflect differences in the behaviors of clinicians and healthcare systems. Our results suggest rating of shared decision making may rely on the surrogate decision maker’s prior experiences and personal characteristics. Our results provide empirical data in support of the commonsensical observation that individuals may interpret the same experience differently based on individual characteristics such as their coping profile. Some individuals may be prone (in this case, adaptive copers) to rate the adequacy of shared decision making more highly than others, even when clinician behavior is invariant. The implication is that different coping profiles (and different personalities or characteristics) will find particular approaches to shared communication more or less satisfying, a further argument for the tailoring of communication and support during the ICU experience. Other researchers have suggested the importance of identifying individual attributes, such as attachment style [44], in support of tailored communication strategies. Our results provide confirmation of the importance of improving the ICU communication experience using a tailored approach.

Future work in ICU settings should explore the implications of these coping profiles for the experience of surrogate decision making and perceived adequacy of shared decision making. Such research would permit further exploration of the reasons individuals with different coping profiles may make different choices in similar clinical situations. Individuals with different coping profiles may make different decisions at least in part because of poorer personal relationships, greater interest in honoring a stated request to not live as a vegetable, greater pessimism about outcomes, or greater skepticism about the role or utility of medical procedures in general. However, further work is needed to examine these and other questions that emerge from this study in the context of clinical interactions in the ICU.

In some prior interventions with ICU families, decision support tools have been used to help family members access information to make decisions [20]. Other studies have used a social worker or staff member as a communication facilitator [45]. Our present results extend this prior work in several important ways. The assessment of “who” the family member is (family member characteristics) identified by our instrument battery may provide a good foundation for identifying which approaches may work best for which groups of individuals. Family members may benefit from receiving information about which methods of support best match their coping profile.

To our knowledge, this is the first study to identify coping profiles among prior and/or potential ICU family members. Within this community sample, we found a striking rate of ICU experience (2/3 of participants), suggesting that many families may enter the ICU with prior ICU experiences, which may color their perception and interaction. For ratings of shared decision making, the gap between adaptive copers and maladaptive or disengaged copers was higher in the subgroup of individuals who had prior ICU experience. The finding that our participants had prior ICU experience both enhances the generalizability of our results and suggests that prior ICU encounters may influence reactions to the current ICU admission.

### Limitations

The study results must be interpreted in light of several limitations. Our respondents were a community sample drawn in part from undergraduate students resulting in a relatively youthful and primarily Caucasian sample of individuals who may not represent current ICU patients and the surrogate decision makers who support them. Notably, two-thirds of our respondents reported a prior ICU experience for themselves or a family member, suggesting that this potential limit to generalizability is not prohibitive. A second limitation is the use of a simulated experience to assess surrogate decision making although it is important to note that similar scenario approaches have been used in other studies of surrogate decision making on behalf of loved ones [46]. In addition, aspects of the specific scenario used here (e.g., requiring participants to imagine making medical decisions on behalf of a loved one with a defined relationship to a sister who was older than our median participant) is also a limitation. We used a multi-faceted approach to identify ICU-relevant individual attributes, with a focus on information processing and mechanisms for coping with stress. Additional individual attributes may be important to include in future studies, especially resilience and propensity for post-traumatic growth [47]. Research is needed in ICU populations to confirm these findings.

### Implications

We identified coping profiles among past or potential ICU family members that were associated with differences in surrogate decision making and ratings of shared decision making during a simulated ICU experience. This suggests that coping profiles may be a useful contribution to future tailoring and support for ICU family members and raises important questions about factors that may influence surrogate decisions and need further study. A next step is to extend this work to a population of families with a loved one currently in the ICU. Our findings suggest that decision support in the ICU should incorporate awareness of the coping profiles and individual attributes of family members, beyond the probabilities and weights typical of many decision analysis approaches.

## Supporting Information

S1 File Appendix A: Vignette-Based Simulated ICU Experience. (DOCX)

S2 File Online Data Supplement: Sensitivity Analysis for Missing Data. (DOCX)
