# Supplementary material for: Preliminary Identification of Coping Profiles Relevant to Surrogate Decision Making in the ICU
Source: PLoS One. 2016 Nov 11;11(11):e0166542. doi: 10.1371/journal.pone.0166542 (PMC5105941; doi:10.1371/journal.pone.0166542)
Supplement: S1 File — (DOCX) [file pone.0166542.s001.docx]

**S1. Appendix A: Vignette-Based Simulated ICU Experience**

**Below you are going to read a scenario and answer questions about it.**

**Joan's Story:**

Imagine that you are Joan's sister/brother, and you have been close your entire life. Joan is 68. She has struggled with obesity and diabetes for 16 years but is still able to enjoy her retirement after teaching junior high school for 30 years. She never had any children, but she has several devoted siblings, including you. You talk often, and she has indicated that she does not want to be kept alive as a vegetable but has not told you anything else about her opinions about medical care. In this scenario, you will accompany Joan through a medical situation, as outlined on the next pages.

**Joan’s Medical Situation**:

Joan developed a chest cold several days ago that progressed to pneumonia, and she was admitted to the hospital yesterday for antibiotics. After a day she is having such a hard time breathing that she is transferred to the intensive care unit. She is breathing fast and is very confused. You are with her at the hospital.

The doctor takes you aside in the hallway outside her hospital room and says, "Her breathing is getting worse. It's bad enough that she will need a machine, called a ventilator, to help her breathe. We think it's the best thing to perform a procedure called 'intubation.' During intubation we insert a breathing tube through her mouth into her windpipe and connect that tube to the ventilator. The ventilator then breathes for her. As with all procedures, we want to obtain your consent to perform this procedure. May we have your consent?"

In situations like this, many people want some additional information in order to respond to a request like this from a physician.

1. Before you decide how to respond to the physician's request, which of the following elements would be MOST important for you to know? Choose the one best answer.

1. How many patients in a similar situation survive their illness.
2. How the procedure of intubation is performed.
3. How uncomfortable intubation and mechanical ventilation will be for Joan.
4. What sort of quality of life Joan will have after her hospital stay.
5. What the doctor would do in this situation if Joan were his/her sister.
6. Nothing. I am comfortable with the physician’s proposal without any additional information.
7. Other (Please describe): ­­­­­­­­____________________________________________

2. Which of the following elements would be SECOND most important for you to know?

1. How many patients in a similar situation survive their illness.
2. How the procedure of intubation is performed.
3. How uncomfortable intubation and mechanical ventilation will be for Joan.
4. What sort of quality of life Joan will have after her hospital stay.
5. What the doctor would do in this situation if Joan were his/her sister.
6. Nothing. I am comfortable with the physician’s proposal without any additional information.
7. Other (Please describe): ­­­­­­­­____________________________________________

3. You included “How many patients in a similar situation survive their illness,” as one of your answers to the above question. Why did you choose this? (Select all that apply.)

1. I would want to know what to expect, regardless of what decision I make.
2. If the chance of survival were too low, I would not allow the doctors to perform the procedure.
3. I would always decide to allow the doctors to perform the procedure, but that’s important information to know, no matter what.

**Continue with Joan’s worsening situation:**

You allow the doctors to perform the procedure, and Joan is intubated. She initially appears to be doing okay but after two days begins to do worse again. She has been uncomfortable on the ventilator, unable to communicate. When they try to awaken her she struggles and thrashes, so they have to put her on more sedatives, which keeps her sleeping. Finally her kidneys start to fail and no longer make urine. The treating doctor greets you beside Joan's bed and says, "Her kidneys are not working right now, and we believe that she needs a machine to clean her blood like a working kidney. This machine is called dialysis. We recommend starting dialysis now. As with all procedures, we want to obtain your consent to perform this procedure. May we have your consent?"

1. Would you.... Choose the one best answer.
2. Ask for more information about what dialysis is.
3. Ask the doctor why Joan’s kidneys are not working.
4. Allow the doctors to start dialysis.
5. Refuse permission to perform dialysis in order to honor Joan’s prior wishes to not be kept alive as a vegetable.
6. Ask for more information about Joan’s chances for recovering from this illness.
