# Supplementary material for: Preliminary Identification of Coping Profiles Relevant to Surrogate Decision Making in the ICU
Source: PLoS One. 2016 Nov 11;11(11):e0166542. doi: 10.1371/journal.pone.0166542 (PMC5105941; doi:10.1371/journal.pone.0166542)
Supplement: S2 File — (DOCX) [file pone.0166542.s002.docx]

**S2. Online Data Supplement: Sensitivity Analysis for Missing Data**

In our primary analysis to identify the coping profiles, we only utilized complete cases. In order to determine whether the missing cases influenced our conclusions, we completed a sensitivity analysis where values were imputed for the incomplete cases. A simple imputation approach was used where predicted values from an appropriate regression (linear, logistic, or ordinal) were used as the imputed values. The relevant covariates in the prediction models were identified using bootstrapped, backwards stepwise regression.

The same clustering approach as the primary analysis was conducted using the imputed data set. Results of the sensitivity analysis were very similar to the original analysis. ODS Figure 1 superimposes the sensitivity analysis (dotted line) on the original analysis (solid line). The significant differences between coping profiles remained the same as the original analysis.
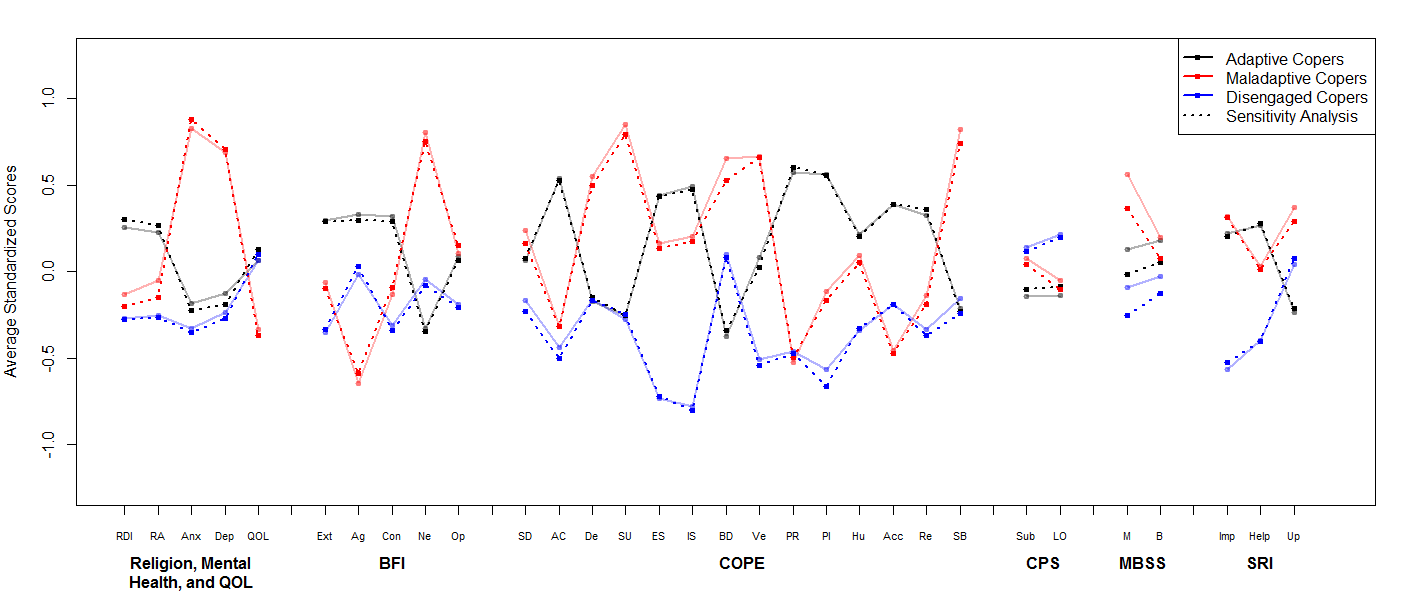


Outcomes by coping profile in the sensitivity analysis were also similar to the original analysis. The top Collaborate score remained significantly different by cluster (p=0.005) with 15% of Adaptive Copers, 7% of Maladaptive Copers, and 3% of Disengaged Copers giving a top score. Disengaged copers were still more likely to refuse dialysis (15%) than Adaptive copers (6%) and Maladaptive copers (7%) [p=0.03].
